# Metabolism Study of Anamorelin, a GHSR1a Receptor Agonist Potentially Misused in Sport, with Human Hepatocytes and LC-HRMS/MS

**DOI:** 10.3390/metabo13080949

**Published:** 2023-08-15

**Authors:** Prince Sellase Gameli, Omayema Taoussi, Giuseppe Basile, Jeremy Carlier, Francesco Paolo Busardò

**Affiliations:** 1 Section of Legal Medicine, Department of Biomedical Sciences and Public Health, Marche Polytechnic University, Via Tronto 10/a, 60126 Ancona, Italy; p.s.gameli@pm.univpm.it (P.S.G.); s1096299@studenti.univpm.it (O.T.); fra.busardo@libero.it (F.P.B.); 2 Department of Trauma Surgery, IRCCS Galeazzi Orthopedic Institute, Via Riccardo Galeazzi 4, 20161 Milan, Italy; giuseppe.basile@unife.it

**Keywords:** anamorelin, ghrelin receptor agonist, doping, metabolism, metabolite prediction, human hepatocyte, liquid chromatography, high-resolution mass spectrometry

## Abstract

Anamorelin, developed for the treatment of cancer cachexia, is an orally active medication that improves appetite and food intake, thereby increasing body mass and physical functioning. It is classified as a growth hormone secretagogue and strictly monitored by the World Anti-Doping Agency (WADA), owing to its anabolic enhancing potential. Identifying anamorelin and/or metabolite biomarkers of consumption is critical in doping controls. However, there are currently no data available on anamorelin human metabolic fate. The aim of this study was to investigate and identify biomarkers characteristic of anamorelin intake using in silico metabolite predictions with GLORYx, in vitro incubation with 10-donor-pooled human hepatocytes, liquid chromatography-high-resolution tandem mass spectrometry (LC-HRMS/MS) analysis, and data processing with Thermo Scientific’s Compound Discoverer. In silico prediction resulted in *N*-acetylation at the methylalanyl group as the main transformation (score, 88%). Others including hydroxylation at the indole substructure, and oxidation and *N*-demethylation at the trimethylhydrazino group were predicted (score, ≤36%). Hepatocyte incubations resulted in 14 phase I metabolites formed through *N*-demethylation at the trimethylhydrazino group, *N*-dealkylation at the piperidine ring, and oxidation at the indole and methylalanyl groups; and two phase II glucuronide conjugates occurring at the indole. We propose four metabolites detected as specific biomarkers for toxicological screening.

## 1. Introduction

The use of performance enhancing agents by athletes to gain a fraudulent edge is a major issue in sporting competitions. In recent years, the abuse of these drugs is prevalent and becoming common among high school athletes and even non-competitive athletic persons, and in some cases may lead to severe and prolonged toxicity and even death [1,2,3]. Substances or agents banned by The World Anti-Doping Agency (WADA) include chemicals that have the potential to enhance performance, pose adverse health consequences and/or violate the spirit of sportsmanship. These substances include anabolic steroids, stimulants, diuretics and narcotics, adrenergic receptor agonists and growth hormones. Synthetic growth hormone secretagogues such as anamorelin and its analogs may increase muscle mass and thus strength, purportedly by targeting ghrelin receptors [4,5].

Ghrelin is a 28-amino-acid hormone obtained from the peptide precursor preproghrelin and produced within the mucosa of the stomach. The discovery of ghrelin and insight on its activity in the hypothalamic melanocortin system in the late 1990s and subsequent research has shown its significance in maintaining homeostasis, enhancing metabolism, and energy expenditure [6,7]. Ghrelin activates its biological effect by binding to the G protein-coupled receptor, growth hormone secretagogue receptor 1 (GHSR1). The posttranslational acylation of ghrelin plays a major role in its interaction with the receptor predominantly located in the pituitary, pancreatic islet, adrenals, thyroid glands, and myocardium. A major fraction of ghrelin binds to GHSR1a receptor with well-characterized neuropeptides that stimulate food intake resulting in its biologic activity, as opposed to the condensed GHSR1b [8].

Anamorelin is an orally active compound that has shown significant efficacy in the treatment of cachexia, after passing phase 3 trials, as compared to its analogs. Cancer cachexia is marked by the disruption of metabolism, loss of appetite and consequently leading to a lean body mass usually occurring in advanced stages. Imbalances in ghrelin and leptin is a prominent feature and intravenously administered ghrelin was recommended, although ghrelin is characterized by a short half-life of approximately 30 min [9]. Although anamorelin has a slightly lower binding affinity as compared to naturally occurring ghrelin, it has a half-life of about 7 to 12 h and peak levels at 2 h after administration and hence is efficacious as compared to ghrelin. Due to its potential to ameliorate dietary consumption and increase body mass, physical function, and strength, it is often misappropriated and/or abused as a performance enhancing drug. Currently, anamorelin is banned by WADA, classified under S2 of the growth hormones secretagogues and their mimetics as part of the prohibit list of substances during sporting events and out of competitions [10]. The side effects of anamorelin have been described and the case of anamorelin-induced fatality resulting from arrhythmias was reported in Japan [11,12].

The detection of anamorelin and its primary metabolites in biological matrices is therefore important in characterizing its consumption and potentially serving as a deterrent against doping and/or abuse. The detection of metabolites is especially crucial to increase detection windows and prevent sample tampering. In an assessment report by the European Medicines Agency (EMA), data on the metabolism of Aldumiz^®^ (anamorelin HCl) were obtained from in vitro incubations with hepatic microsomes from rats, dogs, and humans and cytochrome P450 isoforms; several metabolites were identified after oxygenation and *N*-demethylation, mainly through cytochrome P450 CYP3A4, with minor contributions by CYP2C8 and CYP2D6 [13]. In the same report, it was found that anamorelin and metabolites were mostly excreted in feces with a minor fraction eliminated through urine and expiration when radiolabeled anamorelin was administered to rats and dogs. However, there are no data available on the structure of these metabolites and their relative abundance. In the current study, the potential biomarkers of consumption of anamorelin were investigated through in silico prediction studies, in vitro incubations of anamorelin with human hepatocytes, liquid chromatography high-resolution tandem mass spectrometry (LC-HRMS/MS), and software-aided data mining.

## 2. Materials and Methods

### 2.1. Chemicals and Reagents

We procured pure standards of anamorelin and diclofenac (experimental control) from Toronto Research Chemicals (Toronto, ON, Canada) and Sigma-Aldrich (Milan, Italy), respectively. Stock solutions of the standards were prepared at 1 mg/mL in methanol and stored at −20 °C. LC-MS grade acetonitrile, water, methanol, and formic acid were obtained from Carlo Erba (Cornaredo, Italy). Cryopreserved human hepatocytes (pooled from ten donors), thawing medium, and trypan blue (0.4%) were obtained from Lonza (Basel, Switzerland). Williams’ medium E (WME), *l*-glutamine, and HEPES buffer (2-[4-(2-hydroxyethyl)-1-piperazinyl] ethanesulfonic acid), were also procured from Sigma-Aldrich. HEPES (20 mmol/L) and *l*-glutamine (2 mmol/L) were used in preparing Supplemented Williams’ Medium E (SWM) and stored at 4 °C until incubation.

### 2.2. In Silico Metabolites Prediction

Predicted metabolites of anamorelin were generated using the web-based GLORYx tool (University of Hamburg, Germany), simulating phase I and phase II metabolic reactions in humans using machine learning and site-of-reaction-based prediction models. This was achieved by using the “phase I and II metabolism” option following the generation of Simplified Molecular-Input Line-Entry Specification (SMILES) notation using ACD/ChemSketch (freeware, v. 2020.1.2) [14,15]. Phase I and II metabolites with a corresponding score equal to or greater than 25% were thus considered and incorporated in the inclusion list for LC-HRMS/MS analysis. The predicted metabolic transformations were incorporated in the list of potential reactions for data mining.

### 2.3. Incubation with Pooled Human Hepatocytes

Incubation of anamorelin with cryopreserved hepatocytes was performed as previously described with marginal adjustments were necessary [16]. To briefly summarize, hepatocytes were thawed in 50 mL of thawing medium, centrifuged for 5 min at 50× *g* and the pellets resuspended in 50 mL SWM. Following a second centrifugation, the pellet was again resuspended in 2 mL of SWM after the supernatant was discarded. Cell viability was 87%, as assessed using the trypan blue exclusion method, and the volume of SWM was adjusted to 2 × 10^6^ viable cells/mL.

Next, 250 μL of hepatocyte suspension was gently mixed with 250 μL of 20 μmol/L anamorelin in SWM in a sterile 24-well culture plate using an ArgoLab incubator (Arezzo, Italy). Diclofenac (as a positive control) and negative controls were incubated alongside. The reactions were quenched with 500 μL ice-cold acetonitrile, centrifuged for 10 min at 15,000× *g* and stored at −80 °C for further analysis. All reactions were conducted under physiological conditions.

### 2.4. Sample Preparation

Incubates thawed at room temperature were centrifuged at 15,000× *g* for 10 min. A volume of 100 μL of supernatant was mixed with 100 μL of acetonitrile, then centrifuged again at 15,000× *g* for 10 min. After the supernatants were evaporated to dryness at 37 °C under nitrogen, the resulting residue was reconstituted in 150 μL of mobile phases A and B (MPA: MPB; 95:5, *v*/*v*), vortexed, and transferred into polypropylene microtubes. After centrifugation at 15,000× *g* for 10 min, the supernatants were transferred into glass vials and 10 μL injected into the LC-HRMS/MS system.

### 2.5. LC-HRMS/MS Analysis

LC-HRMS/MS analysis was carried on a DIONEX UltiMate 3000 liquid chromatography coupled with a Q-Exactive quadrupole-Orbitrap hybrid high-resolution mass spectrometer with a heated-electrospray-ionization (HESI) source (Thermo Scientific; Waltham, MA, USA).

#### 2.5.1. Liquid Chromatography Conditions

A Kinetex^®^ Biphenyl column (150 × 2.1 mm, 2.6 μm) from Phenomenex (Torrance, CA, USA) was used, and maintained at 37 °C using 0.1% formic acid in water and 0.1% formic acid in acetonitrile as mobile phase A (MPA) and mobile phase B (MPB), respectively. The chromatographic separation was performed at a flow rate of 0.4 mL/min for a total of 26 min. Gradient elution comprising 95% MPA and 5% MPB was maintained for the first 2 min and MPB gradually increased to 45% and 95% within 16 and 1 min, respectively, and maintained till 22 min. The starting conditions were restored within 0.1 min and maintained till the end of the run.

#### 2.5.2. Mass Spectrometry Conditions

The MS was operated in positive- and negative-ionization modes with HESI settings as follows; spray voltage 3.50 kV, capillary and auxiliary gas temperatures at 300 °C, auxiliary and sheath gas flow rate at 5 and 40 AU, respectively, and S-lens frequency at 40. HESI source setting were optimized after injecting anamorelin standard at 1 μg/mL in MPA:MPB (95:5, *v*/*v*) in the chromatographic conditions of the analysis. The orbitrap was calibrated before analysis, and a lock mass list was used throughout the injections for better mass accuracy. Acquisition of data was from 1 to 22 min in full scan and data-dependent MS/MS mode. Full-scan MS settings included, resolution 70,000 at full width at half maximum (FWHM) at *m/z* 200, automatic gain control (AGC) target 3 × 10^6^, maximum injection time (IT) of 256 ms, and scan range of *m/z* 200–900. Data-dependent MS/MS acquisition settings were as follows; resolution at 17,500, AGC target 2 × 10^5^ with a minimum at 6.5 × 10^2^ to trigger MS/MS (intensity threshold of 10^4^), maximum IT of 64 ms, and isolation window of *m/z* 1.2, normalized collision energy (NCE) of 20 and 45 AU, and dynamic exclusion of 2.0 s. A maximum number of five MS/MS scans were triggered with an inclusion list based on possible metabolic transformations from in silico predictions and speculation for MS/MS acquisition (Supplementary Table S1).

#### 2.5.3. Identification of Metabolites

Data from LC-HRMS/MS were processed with Compound Discoverer (v. 3.1.1.12) from Thermo Scientific using a partial automation by modifying a previously elaborated approach [16]. Briefly, the ions detected in HRMS were compared to a list of theoretical metabolites depending on a list of phase I and phase II transformations based on the in silico predictions as reported in Supplementary Table S2 (intensity threshold of 5 × 10^3^ and mass tolerance of 5 ppm). The HRMS/MS spectra and theoretical elemental composition of the ions were compared to the mzCloud (Counterfeit Drug (Therapeutic), Drugs of Abuse/Illegal Drugs, Sports Doping Drugs, and Therapeutics/Presciption Drugs libraries) and Chemspider (Cayman Chemical and DrugBank libraries) online databases (intensity threshold of 10^5^, HRMS mass tolerance of 5 ppm, and HRMS/MS mass tolerance of 10 ppm). The chromatographic peaks detected in controls with a similar or higher intensity to that of the peaks detected in the samples were included for manual inspection.

## 3. Results

### 3.1. In Silico Prediction

Results from GLORYx freeware predicted twelve first-generation metabolites (pM1–pM12, in decreasing score order). *N*-Acetylation was the main transformation with the highest probability score (88%) occurring at the methylalanyl group. Other transformations predicted included aromatic hydroxylation occurring at the indole, aliphatic hydroxylation at the trimethylhydrazino group, *N*-oxidation at the methylalanyl end, *N*-demethylation at the trimethylhydrazino group, and *N*-glucuronidation at the methylalanyl, with probability scores lower than 40%. Details of in silico predictions including transformation, structure and their respective probability score are attached in Table S3.

### 3.2. Fragmentation Pattern 

**Figure 1:**
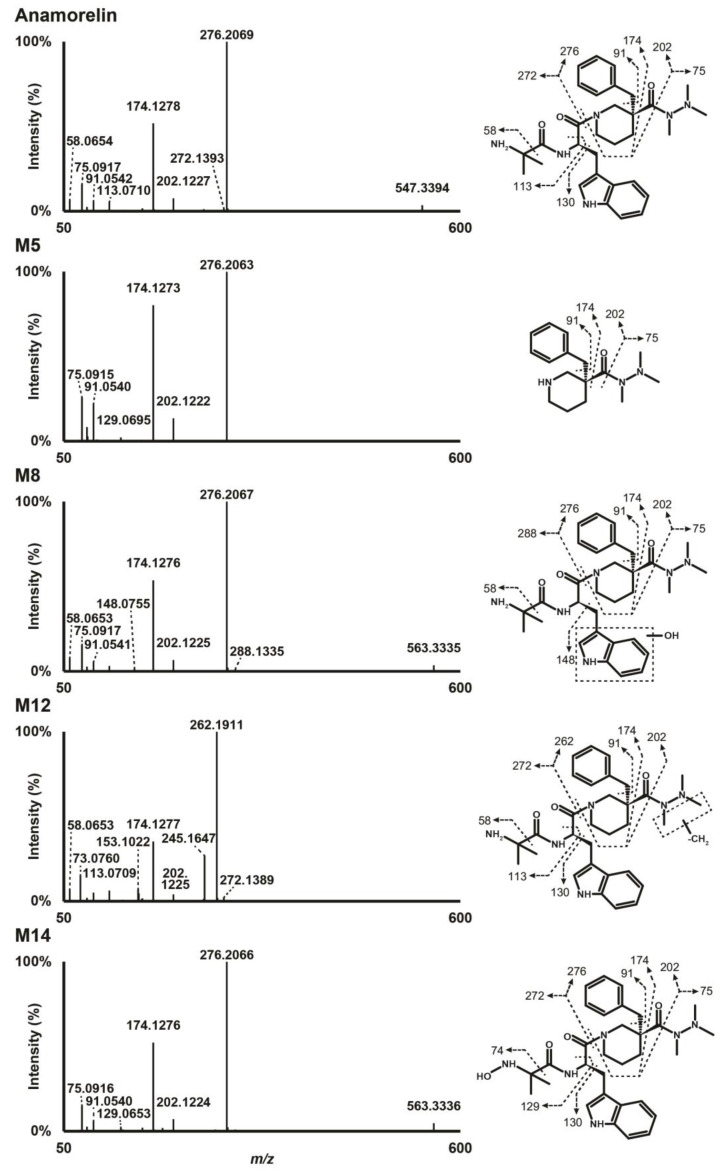
High-resolution tandem mass spectrometry spectra after positive electrospray ionization of anamorelin and major metabolites in human hepatocyte incubations.

Anamorelin was detected at 16.41 min with a signal at *m/z* 547.3395 in positive-polarization mode. The fragmentation pattern showed a base peak at *m/z* 276.2069 produced by cleavage of the carboxamide bond engaged in the piperidine ring. Further α-cleavage from *m/z* 276.2069 at both sides of the carbonyl group produced ions at *m/z* 202.1227 and 174.1278. Other fragments included ions at *m/z* 91.0542, produced by the methylphenyl group, *m/z* 75.0917, by the trimethylhydrazino chain, *m/z* 113.0710, by the methylalanine chain, and *m/z* 130.0649, by the methylindole group. The fragmentation pattern of anamorelin in positive-ionization mode is presented in Figure 1. Negative ionization mode did not yield much meaningful results. Unless otherwise indicated, the ions described in the manuscript refer to their signal in positive-ionization mode.

### 3.3. Metabolite Identification

**Figure 2:**
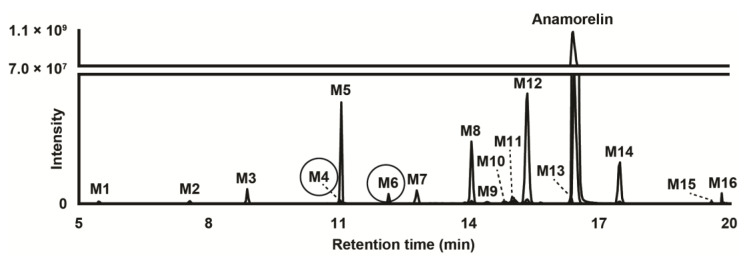
Extracted-ion chromatogram after positive electrospray ionization of anamorelin and metabolites (with phase II metabolites circled) obtained after human hepatocyte incubation for 3 h. Mass tolerance, 5 ppm.

Data from LC-HRMS/MS were automatically processed with Compound Discoverer, which produced 16 metabolites after manual inspection. Fragmentation of major metabolites is displayed in Figure 1, and that of all other metabolites is included in Supplementary Figure S1. The peak area of anamorelin after 3 h incubation with hepatocytes was 5.9 × 10^9^, which was not significantly different from that of incubation after 0 h and might indicate little hepatic metabolism. The Na^+^ adduct of anamorelin had an intensity of 6.1 × 10^8^, i.e., approximately 10% of pseudo-molecular peak area. Fourteen phase I metabolites were detected (Figure 2) following *N*-demethylation; *N*-dealkylation at the piperidine; oxidation at the indole, trimethylhydrazino, and methylalanine groups; carboxylation at the methylalanine; and combination of these transformations. Two additional phase II metabolites resulting from glucuronide conjugation were also identified. Anamorelin metabolites were named M1 to M16 by ascending retention time order. Except *N*-acetylation and *N*-glucuronidation, all in silico predicted metabolites were present after incubation with human hepatocytes. The metabolites detected in the incubations with more than one biotransformations were not predicted considering the initial search parameters. However, in the current study, in silico predictions were only employed to assist in optimizing the LC-HRMS/MS conditions and data mining. The elemental composition, retention time, accurate mass of base-peak ion, LC-HRMS peak area, and diagnostic MS/MS ions of anamorelin and metabolites in positive-ionization mode after 3 h incubation with hepatocytes are reported in Table 1.

#### 3.3.1. N-Demethylation

M12 was the metabolite with the most intense signal, with a base peak at *m/z* 533.3243 detected at 15.33 min and a mass shift of −14.0152 amu suggesting methyl loss (−CH_2_). A M12 main fragment at *m/z* 262.1911 was produced by cleavage of the carboxamide bond engaged in the piperidine ring, and indicated that the transformation was carried by the 3-benzyl-3-[(trimethylhydrazino)carbonyl]-piperidinyl moiety (fragment at *m/z* 276.2069 in parent, minus CH_2_). A fragment at *m/z* 174.1277, also produced by the parent, further indicated that *N*-demethylation occurred at the trimethylhydrazino group. Although minor, a fragment at *m/z* 61.0761 (fragment at *m/z* 75.1277 in parent, minus CH_2_) confirmed the transformation. The exact location of the *N*-demethylation at the trimethylhydrazino group cannot be ascertained in the present analytical conditions. 

M9 was a minor metabolite that eluted at 14.40 min with a −28.0611 amu mass shift from parent, indicating deethylation (−C_2_H_4_) or didemethylation (−2(CH_2_)). M9 had the most abundant fragment at *m/z* 248.1750 indicating that the transformation was carried by the 3-benzyl-3-[(trimethylhydrazino)carbonyl]-piperidinyl moiety (fragment at *m/z* 276.2069 in parent, minus C_2_H_4_). Fragment at *m/z* 174.1273, also produced by the parent, further indicated that *N*,*N*-didemethylation occurred at the trimethylhydrazino group. 

#### 3.3.2. N-Dealkylation (Carboxamide Hydrolysis)

M5 eluting at 11.04 min was produced after hydrolysis of the carboxamide bond engaged in the piperidine group as suggested by the signal at *m/z* 276.2063 indicating a loss of the methylalanyl and methylindole groups (−C_15_H_17_N_3_O_2_). The most intense fragments were at *m/z* 174.1273, 91.0540, and 75.0915 also detected in anamorelin’s fragmentation pattern.

M3 resulted from a combination of *N*-demethylation at the trimethylhydrazino group (−CH_2_) and carboxamide hydrolysis (−C_15_H_17_N_3_O_2_), considering its signal at *m/z* 262.1907 (−285.1488 amu from parent), retention time (8.88 min), and fragmentation pattern.

#### 3.3.3. Oxidation

M8, M11, and M14 were detected at 14.04, 15.05, and 17.46 min, respectively, with a mass shift of +15.9955 amu (±0.3 mmu) from parent, suggesting oxidation (+O). The M8 and M11 fragmentation patterns were similar, with a base peak at *m/z* 276.2067, indicating that the 3-benzyl-3-[(trimethylhydrazino)carbonyl]-piperidinyl moiety was not modified, which was confirmed by major peaks at *m/z* 174.1276 and 75.0917, also detected in the parent. A fragment at *m/z* 58.0653 indicated that the methylalanyl group was also intact, thereby suggesting that the transformation occurred at the methylindole group. A fragment at *m/z* 148.0755 further confirmed the oxidation at the methylindole group, despite the very minute intensity. The lack of water loss pointed towards hydroxylation at the indole group, rather than the methyl chain, both in M8 and M11. M14 was also fragmented to *m/z* 276.2066, 174.1276, and 75.0916, suggesting that no changes occurred at the piperidine and trimethylhydrazino groups. However, M14 also produced *m/z* 130.0648, suggesting that no changes occurring on the indole group, but no fragment at *m/z* 58.0653, suggesting an oxidation at the methylalanyl group. Moreover, the delayed retention time compared to the parent (+1.05 min) indicated *N*-oxidation at the primary amine of the methylalanyl chain.

M2 eluted at 7.56 min with a −257.1539 amu mass shift from anamorelin, indicating *N*-dealkylation at the piperidine ring (−C_15_H_17_N_3_O_2_), oxidation (+O), and dehydrogenation (-H_2_). A base peak at *m/z* 174.1274 and major fragments at *m/z* 202.1223 and 91.0541, all also detected after anamorelin fragmentation, suggested that the trimethylhydrazino group carried the oxidation and dehydrogenation. Considering that aldehydes usually are quickly metabolized to their corresponding alcohol or carboxylic acid, we hypothesized the cyclisation of the trimethylhydrazino group to a more stable methyloxadiazolidinyl group following methyl hydroxylation. Following the same reasoning, we found that M1, eluting at 5.45 min was formed through *N*-dealkylation at the piperidine ring (−C_15_H_17_N_3_O_2_), *N*-demethylation at the trimethylhydrazino group, and oxidation (+O) and cyclisation to an oxadiazolidinyl group (−H_2_) at the dimethylhydrazino group.

M7 and M13 resulted from a combination of *N*-demethylation at the trimethylhydrazino group (−CH_2_) and oxidation (+O), considering their signal at *m/z* 549.3190 (+1.9795 amu from parent). Giving M7 and M13 retention time (12.78, and 16.35 min, respectively) and fragmentation pattern, hydroxylation occurred at the indole group in M7 and *N*-oxidation at the primary amine of the methylalanyl group occurred in M13.

M10 resulted from a combination of hydroxylation at the indole group (+O) and *N*-oxidation at the primary amine of the methylalanyl group (+O), considering its signal at *m/z* 579.3296 (+31.9901 amu from parent), retention time (14.98 min), and fragmentation pattern.

#### 3.3.4. Carboxylation

M16 eluted after anamorelin at 19.81 min with a mass shift of +15.9584 amu, indicating demethylation (−CH_2_), di-oxidation (+2O), and dehydrogenation (−H_2_); it was also detected in negative-ionization mode with an intensity of 9.6 × 10^5^, pointing towards carboxylation. The main fragments at *m/z* 262.1908 and 73.0760 were indicative of *N*-demethylation at the trimethylhydrazino. A minor fragment at *m/z* 130.0649 indicated that the methylindole group was not modified, indicating that carboxylation occurred at a methyl of the methylalanyl group.

M15 was identified with a signal at *m/z* 593.3085 and also eluted after the parent at 19.58 min with a mass shift of +45.9690 amu indicating tri-oxidation (+3O) and dehydrogenation (−H_2_); it was also detected after negative ionization with a signal intensity of 2.0 × 10^5^, pointing towards carboxylation. The M15 fragmentation pattern was poor due to low intensity. However, fragments at *m/z* 276.2068, 174.1274, and 75.0916 indicated that the transformation(s) occurred on the methylalanyl-indole region. 

#### 3.3.5. Glucuronidation

Two conjugated metabolites, M4 and M6 were detected following glucuronidation.

M6 was detected at 12.13 min with a mass shift of −192.0278 amu from parent, signifying oxidation (+O) and glucuronidation (+C_6_H_8_O_6_). M6 fragmentation pattern was similar to that of M8 and M11, indicating hydroxylation at the indole group and glucuronidation. It can be speculated that M6 was produced by glucuronidation of major metabolite M8.

M4 eluted at 11.02 min and was detected with a mass shift of −178.0118 amu from anamorelin, indicating demethylation (−CH_2_), oxidation (+O), and glucuronidation (+C_6_H_8_O_6_). The M4 fragmentation pattern was similar to that of M7, indicating *N*-demethylation at the trimethylhydrazino, hydroxylation at the indole group, and glucuronidation. M4 might be the result of M7 glucuronidation.

## 4. Discussion

The use of drugs to enhance performances is strongly frowned upon, and sensitive methods are usually employed using characteristic markers to identify the consumption of these performance enhancing agents in various matrices [17]. Under physiological conditions, incubation of an illicit compound with liver-derived models effectively simulates hepatic metabolism, producing biomarkers that can be utilized in conjunction with targeted detection of the parent compound by the means of streamlined analytical tools [18]. The use of human hepatocytes is a much-preferred method as compared to liver microsomes, since hepatocytes contain adequate enzymes, cofactors, and transporters for the phase I and II metabolism of xenobiotics [19,20,21,22]. Studies have shown that cryopreservation of human hepatocytes does not meaningfully inhibit or compromise the metabolism of drugs and no difference was observed in freshly isolated and cryopreserved hepatocytes [23,24].

In our study, incubation of anamorelin with cryopreserved human hepatocytes and subsequently analysis with LC-HRMS/MS proved important in the identification and characterization of the main metabolites of anamorelin. We identified a total of 16 metabolites, four as major metabolites including 3-benzyl-3-[(trimethylhydrazino)carbonyl]-1-piperidnyl (M5), hydroxyindole-anamorelin (M8), nor-anamorelin (M12), and *N*-oxoalanyl-anamorelin (M14). We propose these four major metabolites as potential biomarkers of consumption of anamorelin, useful in doping control. To the best of our knowledge, structural analogs of anamorelin such as ipamorelin, tabimorelin, and macimorelin do not produce metabolites akin to the proposed biomarkers. Although minor, glucuronide hydrolysis may improve the signal of oxidated phase I metabolites.

M5 and anamorelin should be chromatographically resolved to avoid potential misidentification, considering that anamorelin was fragmented in the ion source to *m/z* 276.2069 (Figure 2), i.e., the same value as M5 pseudo-molecular ion. With the exception of *N*-dealkylated metabolites, all metabolites and parent compound were accompanied by adducts—especially the Na^+^ adduct—with a relatively high intensity, at the expense of the signal intensity of the corresponding base peaks. When developing an LC-MS method targeting anamorelin and metabolites, special attention should be given to the mobile phase composition and ion source conditions to limit the formation of such adducts.

The lack of human biosamples to corroborate the identified metabolites proposed as biomarkers is a study limitation. Specifically, as a biological matrix of reference, the analysis of urine from authentic anamorelin-positive cases in doping should be performed to better understand the excretion profile of anamorelin and metabolites and refine the present results. This is particularly true as anamorelin and metabolites were shown to be mainly eliminated in feces in rats and dogs [13,25].

## 5. Conclusions

In this study, we identified four potential biomarkers of anamorelin’s metabolic profile, 3-benzyl-3[(trimethylhydrazino)carbonyl]-1-piperidnyl, hydroxyindole-anamorelin, nor-anamorelin, and *N*-oxoalanyl-anamorelin, following in silico prediction, in vitro incubation with hepatocytes, and LC-HRMS/MS analysis and software-assisted data mining. Hepatocyte incubation and data mining resulted in the identification of fourteen phase I and two phase II metabolites following conjugation with glucuronidation. Incorporation of these fragmentation patterns in online databases and libraries and in liquid chromatography-high-resolution mass spectrometry screening methods can prove useful in curbing their misuse. Although human hepatocyte incubations have proved to be a valuable model for predicting drug metabolism in humans, authentic positive biosamples are needed to tentatively confirm our findings. A more detailed in vivo study using urine and/or blood from authentic cases of consumption for doping purposes will be needed to compare with the metabolite biomarkers identified in our study. The detection of anamorelin metabolites found in hepatocyte incubations might aid in identifying authentic positive cases to obtain such samples.

## Figures and Tables

**Table 1:** Elemental composition, retention time, LC-HRMS peak area, and diagnostic product ions of anamorelin and metabolites in positive-ionization mode after 3 h incubation with human hepatocytes. LC-HRMS, liquid chromatography-high-resolution mass spectrometry; RT, retention time; mass tolerance, 5 ppm.

ID	Biotransformation	Elemental Composition	RT, min	[M + H]^+^, *m*/*z*	Mass Error, ∆ppm	Diagnostic Productions, *m*/*z*	Peak Area at T_3h_
M1	*N*-Dealkylation (piperidine) +*N*-Demethylation +Oxidation (*N*-methyl) +Dehydrogenation (*N*-methyl)	C_15_H_21_N_3_O_2_	5.45	276.1709	0.89	82, 91, 174, 202, 276	1.2 × 10^6^
M2	*N*-Dealkylation (piperidine) +Oxidation (*N*-methyl) +Dehydrogenation (*N*-methyl)	C_16_H_23_N_3_O_2_	7.56	290.1856	−2.40	82, 91, 174, 202	5.2 × 10^7^
M3	*N*-Dealkylation (piperidine) +*N*-Demethylation	C_15_H_23_N_3_O	8.88	262.1907	−2.63	91, 153, 174, 202, 245	2.1 × 10^7^
M4	N-Demethylation +Oxidation (indole) +Glucuronidation	C_36_H_48_N_6_O_10_	11.02	725.3513	0.83	73, 91, 174, 245, 262	5.2 × 10^6^
M5	*N*-Dealkylation (piperidine)	C_16_H_25_N_3_O	11.04	276.2063	−2.68	75, 91, 174, 202	3.3 × 10^8^
M6	Oxidation (indole) +Glucuronidation	C_37_H_50_N_6_O_10_	12.13	739.3676	2.00	91, 174, 202, 276, 464	1.5 × 10^7^
M7	*N*-Demethylation +Oxidation (indole)	C_30_H_40_N_6_O_4_	12.78	549.3190	1.13	73, 174, 245, 262, 288	3.0 × 10^7^
M8	Oxidation (indole)	C_31_H_42_N_6_O_4_	14.04	563.3348	1.37	148, 174, 202, 276, 288	1.3 × 10^8^
M9	*N*-Demethylation +*N*-Demethylation	C_29_H_38_N_6_O_3_	14.40	519.3084	1.13	73, 174, 262	6.1 × 10^6^
M10	*N*-Oxidation (dimethylamine) +Oxidation (indole)	C_31_H_42_N_6_O_5_	14.98	579.3296	1.13	75, 148, 174, 202, 276	2.1 × 10^6^
M11	Oxidation (indole)	C_31_H_42_N_6_O_4_	15.05	563.3348	1.37	75, 174, 202, 276, 288	2.6 × 10^7^
M12	*N*-Demethylation	C_30_H_40_N_6_O_3_	15.33	533.3243	1.56	153, 174, 245, 262	3.3 × 10^8^
M13	*N*-Demethylation +*N*-Oxidation (dimethylamine)	C_30_H_40_N_6_O_4_	16.35	549.3190	1.13	73, 174, 187, 245, 262	1.1 × 10^7^
Anamorelin (parent)		C_31_H_42_N_6_O_3_	16.41	547.3395	0.70	75, 91, 174, 202, 272, 276	5.9 × 10^9^
M14	*N*-Oxidation (dimethylamine)	C_31_H_42_N_6_O_4_	17.46	563.3353	2.25	75, 187, 174, 202, 276, 288	1.1 × 10^8^
M15	Oxidation (Indole) +Carboxylation (dimethylamine)	C_31_H_40_N_6_O_6_	19.58	593.3085	0.49	75, 133, 202, 174, 276	4.4 × 10^6^
M16	*N*-Demethylation +Carboxylation (dimethylamine)	C_30_H_38_N_6_O_5_	19.81	563.2979	0.45	73, 130, 174, 245, 262	7.2 × 10^6^

## Data Availability

Raw data were generated at the Department of Biomedical Sciences and Public Health, Marche Polytechnic University. Derived data supporting the findings of this study are available from the corresponding author on request.

## Supplementary Materials

The following supporting information can be downloaded at: https://www.mdpi.com/article/10.3390/metabo13080949/s1, Figure S1: High-resolution tandem mass spectrometry spectra after positive electrospray ionization of anamorelin minor metabolites in human hepatocyte incubations; Table S1: Biotransformation, elemental composition, probability score, and Simplified Molecular-Input Line-Entry Specification (SMILES) of anamorelin metabolites predicted with GLORYx software; Table S2: Transformations for generating anamorelin potential metabolites with Compound Discoverer software for data mining; Table S3: Inclusion list for data-dependent tandem mass spectrometry acquisition.

## References

[1] Wiesing U. (2011). Should Performance-Enhancing Drugs in Sport be Legalized under Medical Supervision?. Sports Med..

[2] Reardon C., Creado S. (2014). Drug abuse in athletes. Subs. Abuse Rehabil..

[3] La Gerche A., Brosnan M.J. (2017). Cardiovascular Effects of Performance-Enhancing Drugs. Circulation.

[4] Min H., Han B., Sung C., Park J.H., Lee K.M., Kim H.J., Kim K.H., Son J., Kwon O.S., Lee J. (2016). LC-MS/MS Method for Simultaneous Analysis of Growth Hormone-Releasing Peptides and Secretagogues in Human Urine. Mass Spectrom. Lett..

[5] World Anti-Doping Agency Prohibited List 2023. https://www.wada-ama.org/sites/default/files/2022-09/2023list_en_final_9_september_2022.pdf.

[6] Wang G., Lee H.M., Englander E., Greeley G.H. (2002). Ghrelin—Not just another stomach hormone. Regul. Pept..

[7] Pradhan G., Samson S.L., Sun Y. (2013). Ghrelin: Much more than a hunger hormone. Curr. Opin. Clin. Nutr. Metab. Care.

[8] Poher A.L., Tschöp M.H., Müller T.D. (2018). Ghrelin regulation of glucose metabolism. Peptides.

[9] Currow D., Maddocks M., Cella D., Muscaritoli M. (2018). Efficacy of Anamorelin, a Novel Non-Peptide Ghrelin Analogue, in Patients with Advanced Non-Small Cell Lung Cancer (NSCLC) and Cachexia—Review and Expert Opinion. Int. J. Mol. Sci..

[10] Uçaktürk E., Başaran A.A., Demirel A.H. (2020). Effect of the Mobile Phase Compositions on the Confirmation Analysis of Some Prohibited Substances in Sport by LC–ESI–MS/MS. Chromatographia.

[11] Okidono Y., Osada J., Otsu K., Kowase S., Aoki H., Yumoto K. (2022). Two cases of wide QRS complex tachycardia caused by anamorelin. J. Cardiol. Cases.

[12] Kojima K., Furukawa S., Ishikawa T., Inoue S. (2023). First case report of anamorelin-induced fatal arrhythmia complicated by sinus arrest and refractory ventricular tachycardia. Hear. Case Rep..

[13] European Medicine Agency Assessment Report–Aldumiz. https://www.ema.europa.eu/en/documents/assessment-report/adlumiz-epar-refusal-public-assessment-report_en.pdf.

[14] Stork C., Embruch G., Šícho M., de Bruyn Kops C., Chen Y., Svozil D., Kirchmair J. (2020). NERDD: A web portal providing access to *in silico* tools for drug discovery. Bioinformatics.

[15] de Bruyn Kops C., Šícho M., Mazzolari A., Kirchmair J. (2021). GLORYx: Prediction of the Metabolites Resulting from Phase 1 and Phase 2 Biotransformations of Xenobiotics. Chem. Res. Toxicol..

[16] Di Trana A., Brunetti P., Giorgetti R., Marinelli E., Zaami S., Busardò F.P., Carlier J. (2021). In silico prediction, LC-HRMS/MS analysis, and targeted/untargeted data-mining workflow for the profiling of phenylfentanyl in vitro metabolites. Talanta.

[17] Birzniece V. (2015). Doping in sport: Effects, harm and misconceptions: Doping in sport. Intern. Med. J..

[18] Ackley D.C., Rockich K.T., Baker T.R. (2004). Metabolic Stability Assessed by Liver Microsomes and Hepatocytes. Optimization in Drug Discovery. Methods in Pharmacology and Toxicology.

[19] Carlier J., Diao X., Huestis M.A. (2018). Synthetic cannabinoid BB-22 (QUCHIC): Human hepatocytes metabolism with liquid chromatography-high resolution mass spectrometry detection. J. Pharm. Biomed. Anal..

[20] Minakata K., Hasegawa K., Nozawa H., Yamagishi I., Saitoh T., Yoshino A., Suzuki M., Kitamoto T., Suzuki O., Watanabe K. (2019). Sensitive quantification of BB-22 and its metabolite BB-22 3-carboxyindole, and characterization of new metabolites in authentic urine and/or serum specimens obtained from three individuals by LC–QTRAP-MS/MS and high-resolution LC–Orbitrap-MS/MS. Forensic Toxicol..

[21] Busardò F.P., Lo Faro A.F., Sirignano A., Giorgetti R., Carlier J. (2022). In silico, in vitro, and in vivo human metabolism of acetazolamide, a carbonic anhydrase inhibitor and common “diuretic and masking agent” in doping. Arch. Toxicol..

[22] Malaca S., Bottinelli C., Fanton L., Cartiser N., Carlier J., Busardò F.P. (2023). α-Methyltryptamine (α-MT) Metabolite Profiling in Human Hepatocyte Incubations and Postmortem Urine and Blood. Metabolites.

[23] Li A.P., Lu C., Brent J.A., Pham C., Fackett A., Ruegg C.E., Silbe P.M. (1999). Cryopreserved human hepatocytes: Characterization of drug-metabolizing enzyme activities and applications in higher throughput screening assays for hepatotoxicity, metabolic stability, and drug-drug interaction potential. Chem. Biol. Interact..

[24] Somers G.I., Lindsay N., Lowdon B.M., Jones A.E., Freathy C., Ho S., Woodrooffe A.J.M., Bayliss M.K., Manchee G.R. (2007). A Comparison of the Expression and Metabolizing Activities of Phase I and II Enzymes in Freshly Isolated Human Lung Parenchymal Cells and Cryopreserved Human Hepatocytes. Drug Metab. Dispos..

[25] Morita-Tanaka S., Yamada T., Takayama K. (2023). The landscape of cancer cachexia in advanced non-small cell lung cancer: A narrative review. Transl. Lung Cancer Res..

