# Supplementary material for: Metabolism Study of Anamorelin, a GHSR1a Receptor Agonist Potentially Misused in Sport, with Human Hepatocytes and LC-HRMS/MS
Source: Metabolites. 2023 Aug 15;13(8):949. doi: 10.3390/metabo13080949 (PMC10456928; doi:10.3390/metabo13080949)
Supplement: Supplementary file 1 [file metabolites-13-00949-s001.zip › AnamorelinMetID_FigureS1_FINAL.pdf]

Figure S1. High-resolution tandem mass spectrometry spectra after positive electrospray ionization of anamorelin minor metabolites in human hepatocyte incubations. Gluc, glucuronide.

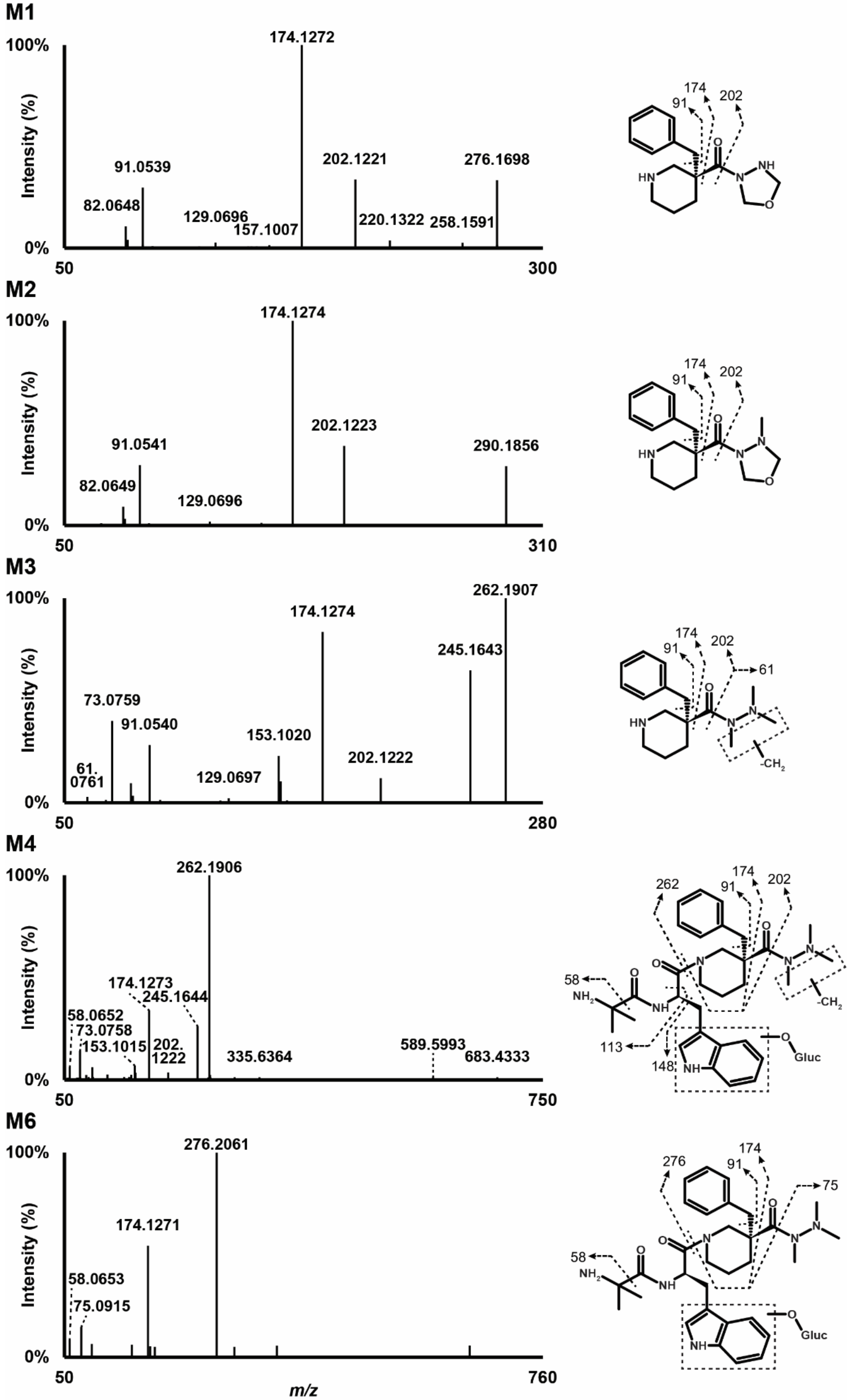

M7

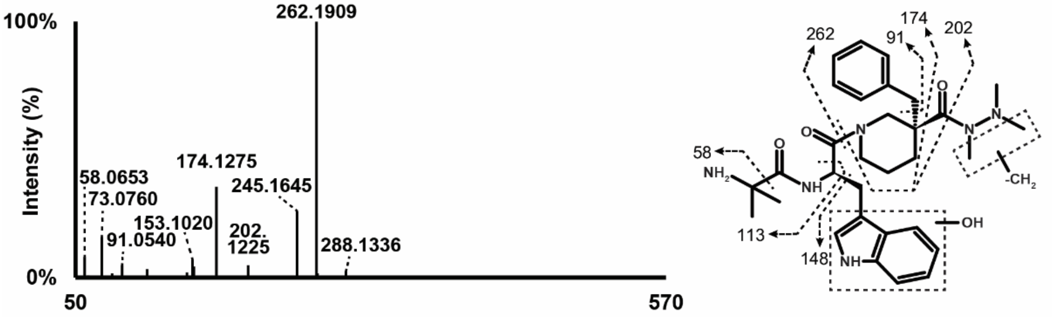

M9

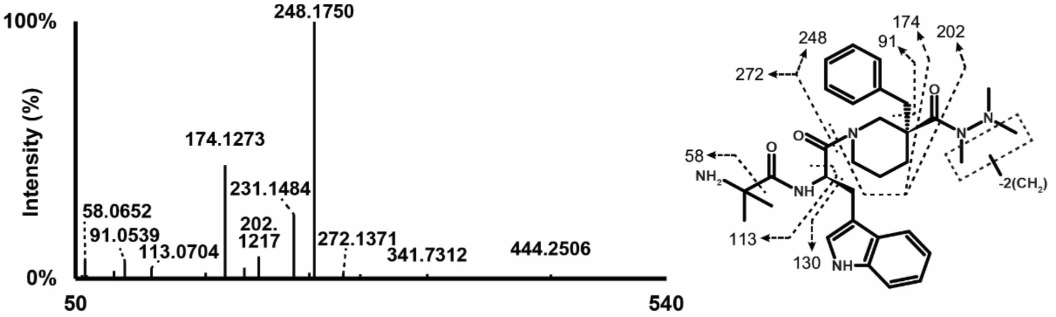

M10

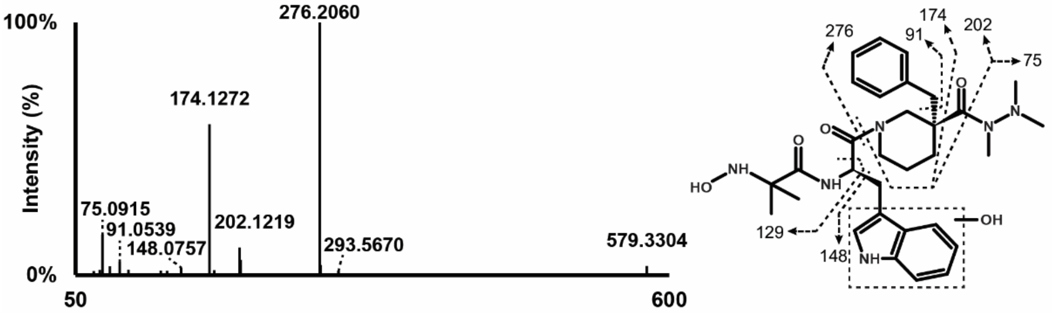

M11

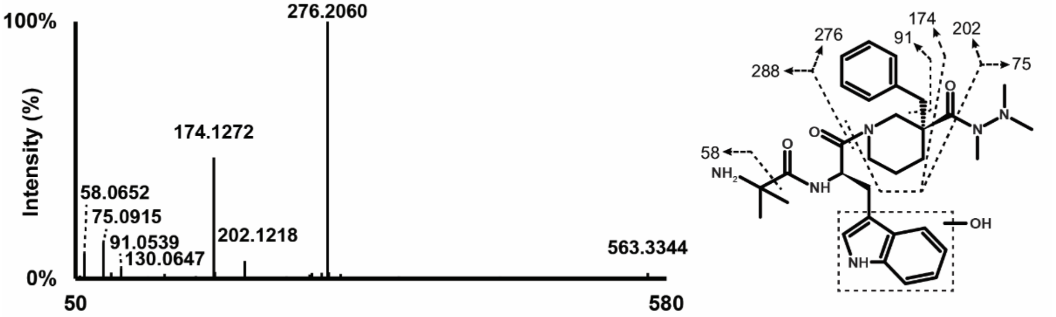

M13

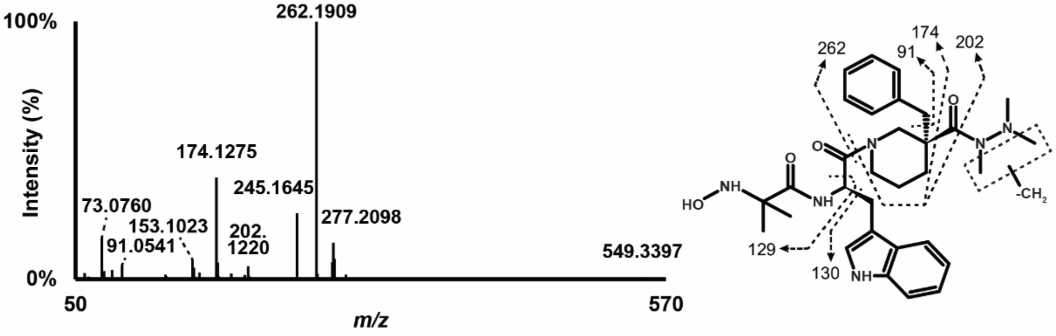

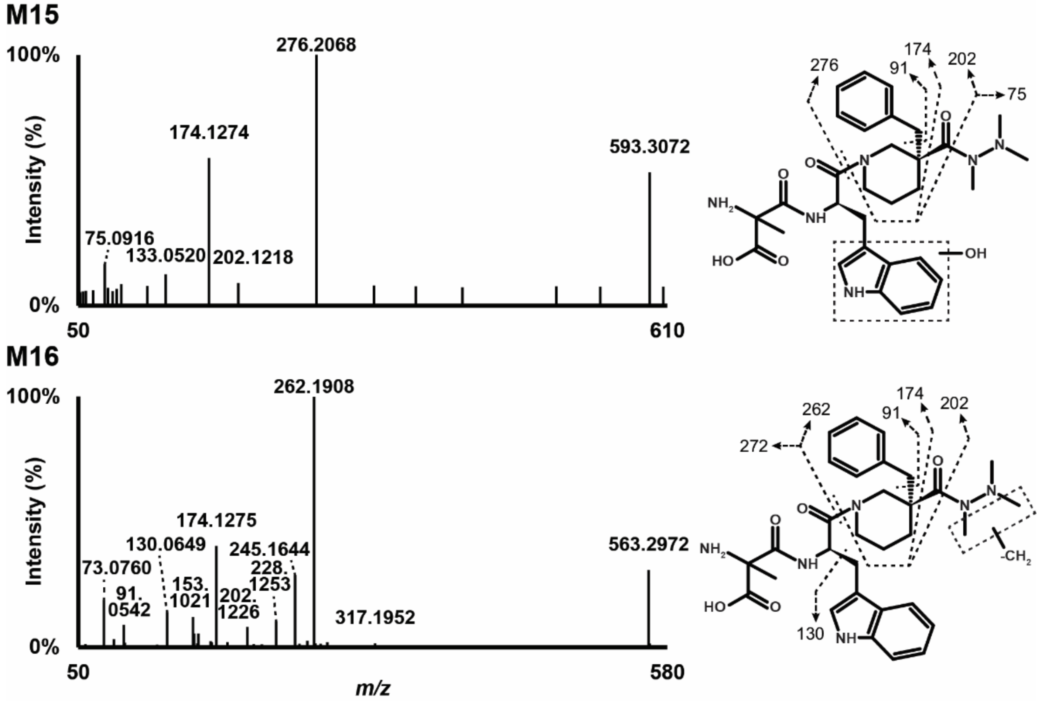
M15
100%
0%
Intensity (%)
276.2068
174.1274
593.3072
75.0916
133.0520
202.1218
50
610
276
174
91
202
75
NH2
NH
HO
O
N
OH
M16
100%
0%
Intensity (%)
262.1908
174.1275
130.0649
245.1644
73.0760
563.2972
91.
0542
153.
1021
228.
1253
202.
1226
317.1952
50
580
m/z
262
272
174
91
202
130
-CH2
