# Supplementary material for: Metabolism Study of Anamorelin, a GHSR1a Receptor Agonist Potentially Misused in Sport, with Human Hepatocytes and LC-HRMS/MS
Source: Metabolites. 2023 Aug 15;13(8):949. doi: 10.3390/metabo13080949 (PMC10456928; doi:10.3390/metabo13080949)
Supplement: Supplementary file 1 [file metabolites-13-00949-s001.zip › AnamorelinMetID_TableS1_FINAL.pdf]

Table S1. Biotransformation, elemental composition, probability score, and Simplified Molecular-Input Line-Entry Specification (SMILES) of anamorelin metabolites predicted with GLORYx software.

**ID**	**Transformation**	**Elemental composition**	**Score (%)**	**SMILES**
Parent		$C_{31}H_{42}N_6O_3$		CC(C)(C(=O)NC(CC1=CNC2=CC=CC=C21)C(=O)N3CCCC(C3)(CC4=CC=CC=C4)C(=O)N(C)N(C)C)N
pM1	*N*-Acetylation	$C_{33}H_{44}N_6O_4$	88	O=C(N2CCCC(C(=O)N(N(C)C)C)(Cc1ccccc1)C2)C(NC(=O)C(NC(=O)C)(C)C)Cc4c3ccccc3nc4
pM2	Aromatic Hydroxylation	$C_{31}H_{42}N_6O_4$	36	O=C(N2CCCC(C(=O)N(N(C)C)C)(Cc1ccccc1)C2)C(NC(=O)C(N)(C)C)Cc4c3ccc(O)cc3nc4
pM3	Aromatic Hydroxylation	$C_{31}H_{42}N_6O_4$	36	O=C(N2CCCC(C(=O)N(N(C)C)C)(Cc1ccccc1)C2)C(NC(=O)C(N)(C)C)Cc4c3cccc(O)c3nc4
pM4	Aromatic Hydroxylation	$C_{31}H_{42}N_6O_4$	36	O=C(N2CCCC(C(=O)N(N(C)C)C)(Cc1ccccc1)C2)C(NC(=O)C(N)(C)C)Cc4c3cc(O)ccc3nc4
pM5	Aromatic Hydroxlation	$C_{31}H_{42}N_6O_4$	36	O=C(N2CCCC(C(=O)N(N(C)C)C)(Cc1ccccc1)C2)C(NC(=O)C(N)(C)C)Cc4c3ccccc3nc4O
pM6	*N*-Demethylation	$C_{30}H_{40}N_6O_3$	30	O=C(N2CCCC(C(=O)N(NC)C)(Cc1ccccc1)C2)C(NC(=O)C(N)(C)C)Cc4c3ccccc3nc4
pM7	Aliphatic Hydroxylation	$C_{31}H_{42}N_6O_4$	30	O=C(N2CCCC(C(=O)N(N(C)CO)C)(Cc1ccccc1)C2)C(NC(=O)C(N)(C)C)Cc4c3ccccc3nc4
pM8	*N*-Oxidation	$C_{31}H_{42}N_6O_4$	30	O=C(N2CCCC(C(=O)N([N+]([O-])(C)C)C)(Cc1ccccc1)C2)C(NC(=O)C(N)(C)C)Cc4c3ccccc3nc4
pM9	*N*-Glucuronidation	$C_{37}H_{50}N_6O_9$	30	O=C(N(N(C)C)C)C5(Cc1ccccc1)CCCN(C(=O)C(NC(=O)C(NC2OC(C(=O)O)C(O)C(O)C2O)(C)C)Cc4c3ccccc3nc4)C5
pM10	*N*-Oxidation	$C_{31}H_{42}N_6O_4$	28	O=C(N2CCCC(C(=O)[N+]([O-])(N(C)C)C)(Cc1ccccc1)C2)C(NC(=O)C(N)(C)C)Cc4c3ccccc3nc4
pM11	*N*-Demethylation	$C_{30}H_{40}N_6O_3$	28	O=C(N2CCCC(C(=O)NN(C)C)(Cc1ccccc1)C2)C(NC(=O)C(N)(C)C)Cc4c3ccccc3nc4
pM12	Aliphatic Hydroxylation	$C_{31}H_{42}N_6O_4$	28	O=C(N2CCCC(C(=O)N(N(C)C)CO)(Cc1ccccc1)C2)C(NC(=O)C(N)(C)C)Cc4c3ccccc3nc4
