# Supplementary material for: Metabolism Study of Anamorelin, a GHSR1a Receptor Agonist Potentially Misused in Sport, with Human Hepatocytes and LC-HRMS/MS
Source: Metabolites. 2023 Aug 15;13(8):949. doi: 10.3390/metabo13080949 (PMC10456928; doi:10.3390/metabo13080949)
Supplement: Supplementary file 1 [file metabolites-13-00949-s001.zip › AnamorelinMetID_TableS2_FINAL.pdf]

Table S2. Transformations for generating anamorelin potential metabolites with Compound Discoverer software for data mining.

Phase I	Desaturation (2H → ø)<br>Dihydrodiol formation (ø → 2H 2O)<br>Ketone formation (O → 2H)<br>Oxidation (ø → O)<br>Oxidative deamination to alcohol (2H N → H O)<br>Oxidative deamination to ketone (2H N → O)<br>Reduction (ø → 2H)
Phase II	Acetylation (H → 2C 3H O)<br>Carnitine conjugation on amine (2H N → 7C 14H N 3O)<br>Cysteine conjugation on dihydrodiol (H O → 3C 6H N 2O S)<br>Cysteine-Glycine conjugation on dihydrodiol (H O → 5C 9H 2N 3O S)<br>Glucuronidation (H → 6C 8H 6O)<br>Glutathionylation on dihydrodiol (H O → 10C 16H 3N 6O S)<br>Glycine conjugation on amine (H → 2C 3H 2O)<br>Methylation (H → C 3H)<br>Sulfation (H → H 3O S)<br>Sulfation on dihydrodiol (H O → H 3O S)<br>Thiol conjugation (H O → H S)
Max # dealkylation	4
Max # Phase II reactions	2
Max # total reactions	5
