# Supplementary material for: Metabolism Study of Anamorelin, a GHSR1a Receptor Agonist Potentially Misused in Sport, with Human Hepatocytes and LC-HRMS/MS
Source: Metabolites. 2023 Aug 15;13(8):949. doi: 10.3390/metabo13080949 (PMC10456928; doi:10.3390/metabo13080949)
Supplement: Supplementary file 1 [file metabolites-13-00949-s001.zip › AnamorelinMetID_TableS3_FINAL.pdf]

Table S3. Inclusion list for data-dependent tandem mass spectrometry acquisition.

Transformation	Elemental composition	$[M+H]^+$ (*m/z*)	$[M-H]^-$ (*m/z*)	Reference transformation
None (anamorelin)	$C_{31}H_{42}N_6O_3$	547.3391	545.3246	Parent
-C -2H	$C_{30}H_{40}N_6O_3$	533.3235	531.3089	*N*-Demethylation
-2C -4H	$C_{29}H_{38}N_6O_3$	519.3078	517.2933	*N*,*N*-Didemethylation
-2C -5H -N	$C_{29}H_{37}N_5O_3$	504.2969	502.2824	*N*-Dealkylation (trimethylhydrazino)
-3C -7H -N	$C_{28}H_{35}N_5O_3$	490.2813	488.2667	*N*-Demethylation + *N*-Dealkylation (trimethylhydrazino)
-3C -8H -2N +O	$C_{28}H_{34}N_4O_4$	491.2653	489.2507	*N*-Demethylation + *N*-Dealkylation (trimethylhydrazino) to COOH
-4C -7H -N -O	$C_{27}H_{35}N_5O_2$	462.2864	460.2718	*N*-Dealkylation (methylalanine)
-15C -17H -3N -2O	$C_{16}H_{25}N_3O$	276.2070	274.1925	*N*-Dealkylation (piperidine)
-16C -22H -2N -O	$C_{15}H_{20}N_4O_2$	289.1659	287.1513	*N*-Dealkylation (piperidine)
-16C -23H -3N	$C_{15}H_{19}N_3O_3$	290.1499	288.1354	*N*-Dealkylation (piperidine) to COOH
+O	$C_{31}H_{42}N_6O_2$	563.3340	561.3195	Oxidation
+2H	$C_{31}H_{44}N_6O_3$	549.3548	547.3402	Reduction
-2H +O	$C_{31}H_{40}N_6O_4$	561.3184	559.3038	Ketone formation
+2O	$C_{31}H_{42}N_6O_5$	579.3289	577.3144	Dioxidation
+4H	$C_{31}H_{46}N_6O_3$	551.3704	549.3559	Direduction
+2H +2O	$C_{31}H_{44}N_6O_5$	581.3446	579.3300	Dihydrodiol formation
+6C +8H +6O	$C_{37}H_{50}N_6O_9$	723.3712	721.3567	Glucuronidation
+6C +10H +6O	$C_{37}H_{52}N_6O_9$	725.3869	723.3723	Reduction + Glucuronidation
+6C +8H +7O	$C_{37}H_{50}N_6O_{10}$	739.3661	737.3516	Oxidation + Glucuronidation
+3O +S	$C_{31}H_{42}N_6O_6S$	627.2959	625.2814	Sulfation
+2H +3O +S	$C_{31}H_{44}N_6O_6S$	629.3116	627.2970	Reduction + Sulfation
+4O +S	$C_{31}H_{42}N_6O_7S$	643.2908	641.2763	Oxidation + Sulfation
+2C +2H +O	$C_{33}H_{44}N_6O_4$	589.3497	587.3351	Acetylation
-H	$C_{30}H_{42}N_6O_3$	545.3235	-	Iminium formation [Charged]
-5C -9H -N -O	$C_{26}H_{33}N_5O_2$	448.2707	446.2561	*N*-Demethylation + *N*-Dealkylation (methylalanine)
-16C -19H -3N -2O	$C_{15}H_{23}N_3O$	262.1914	260.1768	*N*-Demethylation + *N*-Dealkylation (piperidine)
-C -2H +O	$C_{30}H_{40}N_6O_4$	549.3184	547.3038	*N*-Demethylation + Oxidation
-C	$C_{30}H_{42}N_6O_3$	535.3391	533.3246	*N*-Demethylation + Reduction
-C -4H +O	$C_{30}H_{38}N_6O_4$	547.3027	545.2882	*N*-Demethylation + Ketone formation
-6C -12H -2N -O	$C_{25}H_{30}N_4O_2$	419.2442	417.2296	*N*-Dealkylation (trimethylhydrazino) + *N*-Dealkylation (methylalanine)
-17C -22H -4N -2O	$C_{14}H_{20}N_2O$	233.1648	231.1503	*N*-Dealkylation (trimethylhydrazino) + *N*-Dealkylation (piperidine)
-2C -5H -N +O	$C_{29}H_{37}N_5O_4$	520.2918	518.2773	*N*-Dealkylation (trimethylhydrazino) + Oxidation
-2C -3H -N	$C_{29}H_{39}N_5O_3$	506.3126	504.2980	*N*-Dealkylation (trimethylhydrazino) + Reduction
-2C -7H -N +O	$C_{29}H_{35}N_5O_4$	518.2762	516.2616	*N*-Dealkylation (trimethylhydrazino) + Ketone formation
-4C -7H -N	$C_{27}H_{15}N_5O_3$	478.2813	476.2667	*N*-Dealkylation (methylalanine) + Oxidation
-4C -5H -N -O	$C_{27}H_{35}N_5O_2$	464.3020	462.2874	*N*-Dealkylation (methylalanine) + Reduction
-4C -9H -N	$C_{27}H_{33}N_5O_3$	476.2656	474.2511	*N*-Dealkylation (methylalanine) + Ketone formation
-15C -17H -3N -O	$C_{16}H_{29}N_3O_2$	292.2020	290.1874	*N*-Dealkylation (piperidine) + Oxidation
-15C -15H -3N -2O	$C_{16}H_{27}N_3O$	278.2227	276.2081	*N*-Dealkylation (piperidine) + Reduction)
-15C -19H -3N -O	$C_{16}H_{23}N_3O_2$	290.1863	288.1718	*N*-Dealkylation (piperidine) + Ketone formation)
-16C -22H -2N	$C_{15}H_{20}N_4O_3$	305.1608	303.1463	*N*-Dealkylation (piperidine) + Oxidation
-16C -20H -2N -O	$C_{15}H_{22}N_4O_2$	291.1816	289.1670	*N*-Dealkylation (piperidine) + Reduction

-16C -24H -2N	$C_{15}H_{18}N_4O_3$	303.1452	301.1306	*N*-Dealkylation (piperidine) + Ketone formation
-16C -23H -3N +O	$C_{15}H_{19}N_3O_4$	306.1448	304.1303	*N*-Dealkylation (piperidine) to COOH + Oxidation
-16C -21H -3N	$C_{15}H_{21}N_3O_3$	292.1656	290.1510	*N*-Dealkylation (piperidine) to COOH + Reduction
-16C -25H -3N +O	$C_{15}H_{17}N_3O_4$	304.1292	302.1146	*N*-Dealkylation (piperidine) to COOH + Ketone formation
